# Classic Cervical Lymphadenopathy and Ulceration: A Case of Scrofula

**DOI:** 10.4269/ajtmh.24-0634

**Published:** 2025-06-10

**Authors:** Ibrahim Nagmeldin Hassan, Nagmeldin Abuassa

**Affiliations:** Faculty of Medicine, University of Khartoum, Khartoum, Sudan

A 56-year-old man from Sudan presented with multiple firm nodules and enlarged lymph nodes below the ear and on the neck, some of which exhibited ulceration and discharge. The patient had noticed gradual swelling in these areas over the past 3 months; it was initially painless but became increasingly uncomfortable with time. He also reported intermittent fevers, night sweats, and unexplained weight loss. The ulcerating nodules on his neck suggested a chronic, infectious process. The patient’s medical history was unremarkable, with no known history of tuberculosis (TB) or immunosuppression.

**Figure 1.**
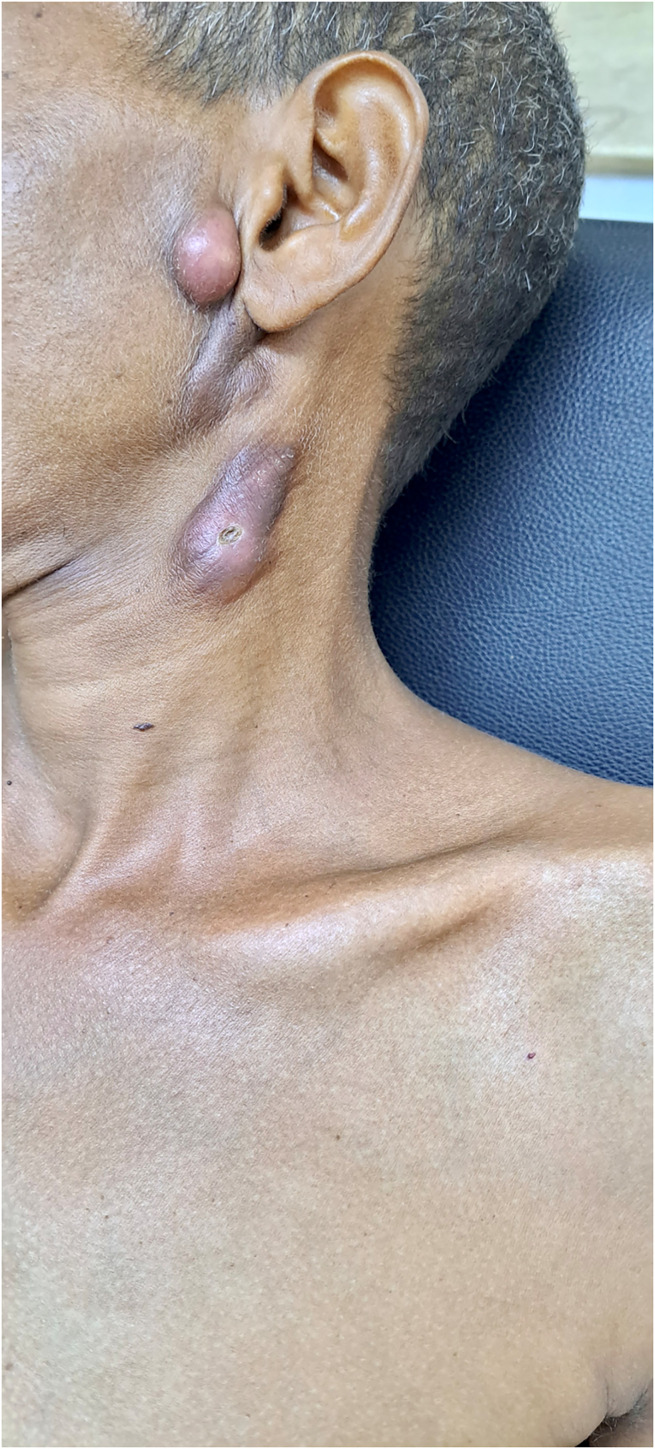
Cervical lymphadenopathy in a patient with scrofula. The image shows multiple enlarged, firm lymph nodes below the ear and along the neck, with some nodes exhibiting surface ulcerations. This characteristic appearance is consistent with tuberculous lymphadenitis.

A clinical examination revealed multiple palpable enlarged cervical lymph nodes, particularly in the posterior triangle of the neck, which is consistent with scrofula (tuberculous lymphadenitis). The largest of the nodes measured ∼3 cm in diameter and exhibited a rubbery consistency. The overlying skin was inflamed, with signs of ulceration and slight purulent discharge. The lesion below the ear was the most prominent, displaying features of abscess formation, with fluctuation noted on palpation (Figure 1).

**Figure 2.**
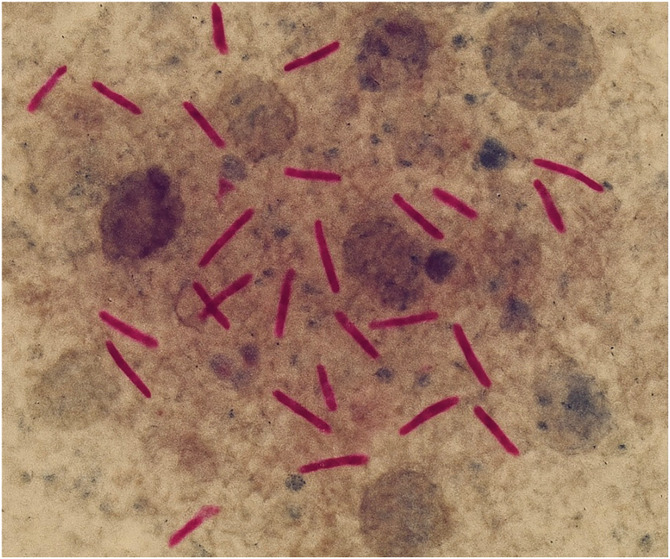
Ziehl–Neelsen stain showing acid-fast bacilli in a lymph node aspirate. The image demonstrates numerous slender, red-colored acid-fast bacilli against a granular, pale background, consistent with *Mycobacterium tuberculosis* infection. This finding supports the diagnosis of tuberculous lymphadenitis (scrofula).

Given the clinical suspicion of TB lymphadenitis, diagnostic tests were conducted. A purified protein derivative test revealed significant induration, and a chest X-ray revealed no active pulmonary TB, suggesting isolated lymphatic involvement. Fine-needle aspiration of the enlarged lymph node was performed, revealing caseating granulomas on cytology, further supporting the diagnosis of tuberculous lymphadenitis (Figure 2). A GeneXpert (Cepheid, Sunnyvale, CA) test confirmed the presence of *Mycobacterium tuberculosis* (*M. tuberculosis*) in the aspirate, and the patient was started on anti-TB therapy (ATT), with a regimen consisting of rifampin, isoniazid, pyrazinamide, and ethambutol.

Although *Mycobacterium bovis*—a strain of the *M. tuberculosis* complex—can cause scrofula through the ingestion of unpasteurized milk from infected livestock, the patient denied any such exposure. Given the high prevalence of *M. tuberculosis* in Sudan and the absence of zoonotic exposure, we believe that the causative agent in this case was *M. tuberculosis*. Unfortunately, because of limited resources, species-level identification was not performed.

The patient responded well to ATT, with gradual resolution of the lymphadenopathy and improved systemic symptoms after a few weeks of treatment. A follow-up examination after 2 months of therapy revealed a significant reduction in the size of the lymph nodes and healing of the ulcerated areas.

Scrofula remains a common presentation of extrapulmonary TB, particularly in regions with high TB prevalence, such as Sudan. Early diagnosis and the prompt initiation of ATT are critical for preventing complications such as fistula formation or extensive soft tissue involvement.^1^ This case highlights the importance of recognizing the clinical features of tuberculous lymphadenitis and considering TB as a differential diagnosis in patients with chronic lymphadenopathy, especially in endemic areas.
